# *ANGPTL3* gene variants in subjects with familial combined hyperlipidemia

**DOI:** 10.1038/s41598-021-86384-y

**Published:** 2021-03-26

**Authors:** A. M. Bea, E. Franco-Marín, V. Marco-Benedí, E. Jarauta, I. Gracia-Rubio, A. Cenarro, F. Civeira, I. Lamiquiz-Moneo

**Affiliations:** 1 Unidad de Lípidos, IIS Aragón, CIBERCV, Hospital Universitario Miguel Servet, Avda. Isabel La Católica 1-3, 50009 Zaragoza, Spain; 2 Universidad de Zaragoza, Zaragoza, Spain; 3 Instituto Aragonés de Ciencias de la Salud (IACS), Zaragoza, Spain

**Keywords:** Disease genetics, Genetic predisposition to disease

## Abstract

Angiopoietin-like 3 (ANGPTL3) plays an important role in lipid metabolism in humans. Loss-of-function variants in *ANGPTL3* cause a monogenic disease named familial combined hypolipidemia. However, the potential contribution of *ANGPTL3* gene in subjects with familial combined hyperlipidemia (FCHL) has not been studied. For that reason, the aim of this work was to investigate the potential contribution of ANGPTL3 in the aetiology of FCHL by identifying gain-of-function (GOF) genetic variants in the *ANGPTL3* gene in FCHL subjects. *ANGPTL3* gene was sequenced in 162 unrelated subjects with severe FCHL and 165 normolipemic controls. Pathogenicity of genetic variants was predicted with PredictSNP2 and FruitFly. Frequency of identified variants in FCHL was compared with that of normolipemic controls and that described in the 1000 Genomes Project. No GOF mutations in *ANGPTL3* were present in subjects with FCHL. Four variants were identified in FCHL subjects, showing a different frequency from that observed in normolipemic controls: c.607-109T>C, c.607-47_607-46delGT, c.835+41C>A and c.*52_*60del. This last variant, c.*52_*60del, is a microRNA associated sequence in the 3′UTR of *ANGPTL3*, and it was present 2.7 times more frequently in normolipemic controls than in FCHL subjects. Our research shows that no GOF mutations in *ANGPTL3* were found in a large group of unrelated subjects with FCHL.

## Introduction

Angiopoietin-like 3 (ANGPTL3) is a 70 kDa-secreted (54 kDa before glycosylation) protein, mainly expressed in the liver, discovered by Conklin et al. in 1999^1^. ANGPTL3 is an endogenous inhibitor of lipoprotein lipase (LPL) and endothelial lipase (EL)^2,3^. Different studies in families with hypolipemia and in general population have reported that loss-of-function (LOF) variants in *ANGPTL3* gene are associated with decreased plasma levels of triglycerides (TG), low-density lipoprotein cholesterol (LDLc) and high-density lipoprotein cholesterol (HDLc)^4^. The N-terminal domain of ANGPTL3 containing residues from 17 to 207 is responsible for the increased plasma TG levels in mice. Loss of this region prevents the inhibition of LPL^5^ and EL^3^ by ANGPTL3. Recently, the inhibition of ANGPTL3 with a human monoclonal antibody against ANGPTL3 (evinacumab) in dyslipidemic mice and in healthy volunteers caused a dose-dependent placebo-adjusted reduction in fasting TG levels of up to 76% and LDLc levels of up to 23%^4^. Therefore, ANGPTL3 has been considered a potent modulator of TG^2^ and supports an important role of ANGPTL3 in lipid metabolism in humans.

In addition, new evidence sustains a possible role of ANGPTL3 in the progression of atherosclerosis through a lipid-independent mechanism^6^. Carriers of LOF mutations in *ANGPTL3* associated a 34% decrease in cardiovascular events^7^ and ANGPTL3 plasma concentration was associated with arterial wall thickness in humans^8^. Moreover, a decreased expression of ANGPTL3 in apolipoprotein E null (apoE-/-) mice was protective in the development of atherosclerosis^9^.

Familial combined hyperlipidemia (FCHL) is a common and complex inherited disorder of lipid metabolism with important environmental influences^10^. FCHL is characterized by elevated very low-density lipoprotein (VLDL) and/or LDL concentrations, low HDLc levels^11^, and frequently, reduced LPL activity^12^. The FCHL genetic background is mostly polygenic and associated with the variation in at least 35 different genes, including genes related to metabolic disorders such as obesity, peripheral insulin resistance, type 2 diabetes, hypertension and metabolic syndrome^13,14^. However, FCHL is a genetically heterogeneous syndrome and monogenic and oligogenic cases have been also described^15–17^. Subjects with FCHL have high predisposition to develop premature cardiovascular disease (CVD). Actually, FCHL is the most common genetic lipid abnormality found in subjects with premature coronary heart disease^18^. The FCHL phenotype is quite similar to that observed after ANGPTL3 administration in mice. However, the potential involvement of the *ANGPTL3* gene in FCHL has not been previously analysed in contrast with the major role of a loss-of-function mutation in *ANGPTL3* in the opposite situation, familial combined hypolipidemia^19,20^. Therefore, the aim of this study was to identify gain-of-function (GOF) genetic variants in *ANGPTL3* gene in FCHL subjects and to establish the potential contribution of ANGPTL3 in the aetiology of FCHL.

## Material and methods

### Subjects

#### Cases

**Figure 1:**
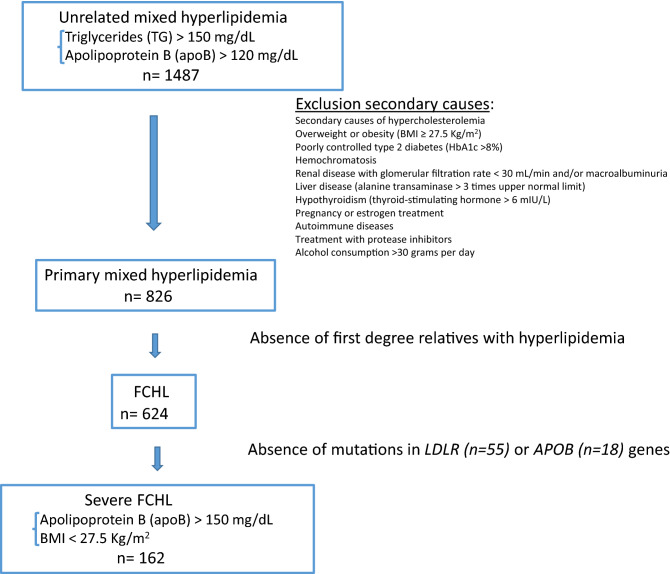
Flow chart of subject selection process.

A total of 162 unrelated subjects, aged 23 to 82, with the clinical diagnosis of severe FCHL from Lipid Unit at Hospital Universitario Miguel Servet, Zaragoza, Spain, were selected for this study. Severe FCHL included: LDLc and TG > 90th percentile adjusted for age and sex, apolipoprotein B (apoB) > 150 mg/dL, body mass index (BMI) < 27.5 kg/m^2^ and at least one first-degree family member with mixed hyperlipidemia. Clinical exclusion criteria were: secondary causes of hypercholesterolemia including significant overweight or obesity (BMI ≥ 27.5 kg/m^2^), poorly controlled type 2 diabetes (HbA1c > 8%), hemochromatosis, renal disease with glomerular filtration rate < 30 mL/min and/or macroalbuminuria, liver disease (alanine transaminase > 3 times upper normal limit), hypothyroidism (thyroid-stimulating hormone > 6 mIU/L), pregnancy or estrogen treatment, autoimmune diseases, treatment with protease inhibitors and alcohol consumption > 30 g per day (Fig. 1).

Most of the subjects included in this work had been studied previously to discard severe genetic defects in the genes regulating the LPL pathway^21^. Subjects with *LDLR, APOB* or *PCSK9* functional mutations causing familial hypercholesterolemia (FH) and subjects with dysbetalipoproteinemia and the *APOE*2/2 genotype were excluded from the study. The lipid phenotype of FH and dysbetalipoproteinemia may overlap with FCHL and with this approach both genetic hyperlipidemias were ruled out to avoid confusion with FCHL.

#### Controls

We selected 165 consecutive normolipemic, unrelated subjects, aged 20–79, who underwent a medical visit at our hospital as control group. Exclusion criteria for control subjects were personal or parental history of premature cardiovascular disease (before 55 years in men and 65 years in women) or personal or parental dyslipidaemia, current acute illness, or use of drugs that might influence glucose or lipid metabolism.

In all subjects, clinical and analytical variables were registered, including personal and familial risk factors, history of cardiovascular disease and intake of drugs affecting intestinal or lipid metabolism.

All experimental protocols were approved by our local ethical committee (Comité Ético de Investigación Clínica de Aragón, CEICA, Zaragoza, Spain). Informed consent was obtained from all subjects before participating in the protocol. Samples from patients included in this study were provided by the Biobank of the Aragon Health System (PT17/0015/0039), integrated in the Spanish National Biobanks Network, and they were processed following standard operating procedures with the appropriate approval of the Ethics and Scientific Committees.

### Biochemical analysis

Ethylenediaminetetraacetic acid (EDTA) plasma and serum samples were collected from all participants after at least 10 h fasting, without lipid-lowering drugs for > 5 weeks, to obtain baseline biochemical characteristics. Total cholesterol (TC) and TG measurements were performed with commercially available diagnostic kits (Boehringer Mannheim, Germany), in a laboratory participating in a lipid standardisation programme. HDLc was measured directly by an enzymatic reaction using cholesterol oxidase (UniCel DxC 800; Beckman Coulter Inc., Brea, California, USA). ApoA1, apoB and lipoprotein(a)^22^ were determined by IMMAGE kinetic immunonephelometry (Beckman Coulter Inc., Brea, California, USA). LDLc was calculated using the Friedewald’s formula^23^. All methods were carried out in accordance with guidelines and regulations of Spanish Society of Clinical Biochemistry.

### Genetic analysis

DNA was isolated from EDTA blood samples using the KingFisher Duo Prime System (Thermo Fisher Scientific). A previously described protocol for sequencing the exon 4 of *APOE* gene^24^ was used for disclosing *APOE*2/2 genotype or functional mutations in exon 4 of the *APOE* gene in order to rule out carrier subjects. Moreover, *LDLR*, *APOB* and *PCSK9* genes were analysed for functional mutations with Lipochip platform (Progenika Grifols, Spain)^25^ in order to rule out subjects with any pathogenic mutation in these genes.

*ANGPTL3* gene (NM_014495.4) was amplified in 7 fragments by polymerase chain reaction with primers showed in Table 1. Each amplified fragment comprised the corresponding exon and its 5′ and 3′ flanking sequences, including intron–exon boundaries. After purification with ExoSap-IT (USB), amplified fragments were sequenced by the Sanger method^26^ using the BigDye 3.1 sequencing kit (Applied Biosystems) in an automated ABI 3500xL sequencer (Applied Biosystems). DNA sequences were analysed using Variant Reporter software (Applied Biosystems).

**Table 1:** Primers and conditions used for *ANGPTL3* amplification and sequencing.

*ANGPTL3*	Primer sequence 5′ → 3′	Annealing temperature (°C)	Product size (bp)
Fragment 1	F: CCTTACCTTTTCTGGGCAA	51.5	821
	R: AAATGCAAATTTTCAGTGTTTTCA		
Fragment 2	F: GCTGGGCTTTTTCTTTTAATTG	51	496
	R: CTTCAGAGCCTGCAATTTT		
Fragment 3	F: CCGACCAATGTCTGCTTTTT	51	555
	R: TCAAGTCCATATTTGTATTTCTCTG		
Fragment 4	F: TCCAGACTGGTGATAGAACAAG	53.5	597
	R: GGCAATTAATGAATTTTGGCATAGT		
Fragment 5	F: TCTCCTTTTCCTCTAAAATAATCTGAA	52.5	596
	R: TGATCATTGTAAGCCGTGG		
Fragment 6	F: ATGCATTATAGAAAGGATAATCAGACT	52.5	700
	R: GAGGAAGATTAGAGGTAAAATACCTG		
Fragment 7	F: ACCTCTAATCTTCCTCAGATTTTC	51	599
	R: TTTTGATTGAGAAATGTAAACGGTA		

Each amplified fragment comprises the corresponding exon and its 5′ and 3′ flanking sequences, including intron–exon boundaries.

*F* forward, *R* reverse.

To evaluate the pathogenicity of new identified genetic variants, we used PredictSNP2^27^. The effect of variants in potential splicing sites was predicted with FruitFly^28^. To compare the frequency of identified variants with that of the general population, we compiled the allele frequencies of identified variants from the 1000 Genomes Project^29^ and genome aggregation data base (gnomAD)^30^. ClinVar database was used for additional information about genomic variation and its relationship to human health^31^. Finally, information about microRNAs was obtained from PolymiRTS Database 3.0^32^. All methods were carried out in accordance with guidelines and regulations of Spanish Society of Human Genetics.

### Statistical analysis

Analyses were performed using statistical computing software R version 3.5.0^33^. The level of significance was set at *P* < 0.05. The distribution of the variables was analysed by the Shapiro test. Quantitative variables with a normal distribution were expressed as mean ± standard deviation and were analysed by the Student *t* test. Variables with a skewed distribution were expressed as medians and interquartile ranges and were analysed with the Mann–Whitney *U* test. Qualitative variables were expressed as percentages and were analysed by the Chi squared test.

## Results

### Study subjects

The main clinical and biochemical characteristics of both studied groups (162 FCHL subjects and 165 normolipemic controls) are presented in Table 2. FCHL subjects showed higher predominance of males (60.5%) and were significantly older than normolipemic subjects (*P* = 0.022 and *P* < 0.001, respectively). Compared with normolipemic controls, FCHL subjects had significantly higher values of BMI, TC, TG, LDLc, apoB and lipoprotein(a) (*P* < 0.001, *P* < 0.001, *P* < 0.001, *P* < 0.001, *P* < 0.001 and *P* = 0.003, respectively). FCHL subjects presented higher prevalence of hypertension, type 2 diabetes and CVD than normolipemic subjects (*P* = 0.009, *P* = 0.001 and *P* = 0.016, respectively). The *APOE* genotype distribution was homogenous between both cohorts, being E3/3 genotype the most frequent in both groups, although E3/2 genotype had a lower frequency in FCHL subjects (5.56%) in contrast to normolipemic subjects (15.2%).

**Table 2:** Clinical and biochemical characteristics in FCHL subjects and normolipemic controls.

	FCHL subjects n = 162	Normolipemic controls n = 165	*p*
Men, n (%)	98 (60.5)	78 (47.3)	0.022
Age (years)	50.4 ± 11.4	38.5 ± 14.7	< 0.001
Body Mass Index (kg/m^2^)	25.6 (24.2–26.5)	23.6 (21.4–26.6)	< 0.001
Total Cholesterol (mg/dL)	312 ± 36.1	170 ± 21.0	< 0.001
Triglycerides (mg/dL)	277 (232–373)	64.0 (49.0–93.0)	< 0.001
LDL cholesterol (mg/dL)	204 (183–230)	108 (91.8–117)	< 0.001
HDL cholesterol (mg/dL)	48.5 ± 12.0	55.7 ± 11.4	0.015
Apolipoprotein A1 (mg/dL)	147 ± 25.0	147 ± 27.4	0.930
Apolipoprotein B (mg/dL)	167 (165–190)	83.0 (72.0–91.0	< 0.001
Lipoprotein(a), (mg/dL)	39.1 (10.3–80.8)	16.2 (7.79–44.5)	0.003
Glucose (mg/dL)	93.0 (86.0–103)	85.0 (80.0–92.0)	< 0.001
HbA1c (%)	5.50 (5.30–5.80)	5.20 (5.00–5.40)	< 0.001
Type 2 diabetes, n (%)	13 (8.02)	2 (1.21)	0.009
Hypertension, n (%)	30 (18.5)	10 (6.06)	0.001
Cardiovascular disease, n (%)	7 (4.32)	0	0.016
**Tobacco, n (%)**			
Non smoker	51 (31.5)	96 (58.1)	< 0.001
Smoker	70 (43.2)	31 (18.8)	
Former smoker	40 (24.7)	28 (16.7)	
**Apolipoprotein E genotype, n (%)**			
E3/3	113 (69.8)	109 (66.1)	0.035
E3/2	9 (5.56)	25 (15.2)	
E2/2	0	0	
E3/4	31 (19.1)	25 (15.2)	
E4/4	6 (3.70)	2 (1.21)	
E2/4	3	4	

Quantitative continuous variables are expressed as mean ± standard deviation or median [percentile 25–75]. Student’s t or Mann–Whitney tests were used to assess differences between two groups. Quantitative categorical variables are expressed as n (%) and statistical differences were assessed by Chi-squared.

### *ANGPTL3* genetic variants

Table 3 shows all variants in the *ANGPTL3* gene identified in both groups. A total of 16 genetic variants, four of them not previously described, were identified by sequencing analysis. Only four of them (c.607-109T>C, c.607-47_607-46delGT, c.835+41C>A and c.*52_*60del) presented significantly different allele frequency in normolipemic group than in FCHL subjects (*P* = 0.020, *P* = 0.031, *P* = 0.043 and *P* < 0.001, respectively). Out of the 16 variants, seven variants were located in the coding region (c.379C>T, c.565T>C, c.961T>A, c.1003T>C, c.1028A>G, c.1089T>G and c.1122G>A), and three of them were missense variants: p. (Leu127Phe), p.(Tyr321Asn) and p.(His343Arg), but only p.(Leu127Phe) was described as deleterious by bioinformatics analysis. The other four variants located in the coding region, p.(Leu189Leu), p.(Leu335Leu), p.(Val363Val) and p.(Pro374Pro) were synonymous variants. Seven variants were located in the intronic region, c.496-88T>G, c.607-120A>G, c.607-109T>C, c.607-47_607-46delGT, c.835+41C>A, c.1198+111G>A and c.1198+140T>C. All of them were described as benign or not splicing change affected by the bioinformatics analysis. Nevertheless, three of them, c.607-109T>C, c.607-47_607-46delGT and c.835+41C>A, presented significantly higher allele frequency in FCHL subjects than in the normolipemic group. Finally, two variants were located in the 3′UTR, c.*52_*60del and c.*76T>G. One of them, c.*52_*60del, showed significantly higher allele frequency in the normolipemic group than in FCHL subjects.

**Table 3:** Frequency and bioinformatics analysis of identified variants in *ANGPTL3* in FCHL cases and controls.

Variant	Location	Nucleotide change	Protein change	Bioinformatics analysis		Allele frequency in the general population		Allele frequency in our study			ACMG classification^e^	MicroRNAs^f^
				PredictSNP2^a^ (probability)	FruitFly^b^	GnomAD^c^	1000 Genomes Project^d^	Normolipemic subjects	FCHL subjects	*p*		
rs72649573	Exon 1	c.379C>T	p.(Leu127Phe)	Deleterious (82%)	NA	0.00711	0.0020	0.000	0.003	0.313	Benign^g^	NR
–	Intron 1	c.496-88T>G	NA	Neutral (88%)	Not splicing change	–	–	0.000	0.003	0.313	–	–
rs111414963	Exon 2	c.565T>C	p.(Leu189Leu)	Neutral (88%)	Not splicing change	0.00025	0.0008	0.003	0.000	0.313	Likely benign	NR
rs531071581	Intron 2	c.607-120A>G	NA	Neutral (88%)	Not splicing change	0.00013	0.0006	0.000	0.003	0.313	–	NR
rs72649576	Intron 2	c.607-109T>C	NA	Neutral (88%)	Not splicing change	0.01079	0.0042	0.024	0.003	0.020	–	NR
rs72649577	Intron 2	c.607-47_607-46delGT	NA	Neutral (88%)	NA	0.02222	0.0136	0.022	0.003	0.031	–	NR
rs185472483	Intron 3	c.835+41C>A	NA	–	Not splicing change	0.00032	0.0006	0.000	0.012	0.043	–	NR
rs747725081	Exon 6	c.961T>A	p.(Tyr321Asn)	Neutral (88%)	NA	NR	NR	0.000	0.003	0.313	–	NR
rs12563308	Exon 6	c.1003T>C	p.(Leu335Leu)	Neutral (88%)	NA	0.03550	0.0559	0.003	0.003	0.989	VUS^g^	NR
rs199555921	Exon 6	c.1028A>G	p.(His343Arg)	Neutral (89%)	NA	0.00016	NR	0.003	0.000	0.321	–	NR
rs763259225	Exon 6	c.1089T>G	p.(Val363Val)	Neutral (96%)	NA	NR	NR	0.003	0.000	0.321	–	NR
rs145086916	Exon 6	c.1122G>A	p.(Pro374Pro)	Neutral (96%)	NA	0.00077	0.0006	0.003	0.000	0.321	–	NR
rs72651034	Intron 6	c.1198+111G>A	NA	–	Not splicing change	NR	NR	0.003	0.003	0.989	–	NR
rs908541128	Intron 6	c.1198+140T>C	NA	–	Not splicing change	0.000	0.000	0.003	0.000	0.321	–	NR
rs34483103	3′UTR	c.*52_*60del	NA	–	NA	0.33531	0.3484	0.276	0.102	< 0.001	–	hsa-miR-151a-3p hsa-miR-7702
–	3′UTR	c.*76T>G	NA	–	NA	–	–	0.000	0.003	0.313	–	–

*NR* not reported, *NA* not applicable, *VUS* variant of uncertain significance.

^a^PredictSNP2 uses CADD, DANN, FATHMM and Funseq2 as predictors.

^b^FruitFly. New prediction score 0.87 (wild type score 0.89).

^c^GnomAD. https://gnomad.broadinstitute.org/

^d^1000 Genomes Project Consortium, Abecasis GR, Auton A, Books LD et al. An integrated map of genetic variation from 1092 human genomes. Nature 2012;491:56–65.

^e^Richards S, Aziz N, Bale S, Bick D, Das S, Gastier-Foster J, Grody WW, Hegde M, Lyon E, Spector E, Voelkerding K, Rehm HL; ACMG Laboratory Quality Assurance Committee. Standards and guidelines for the interpretation of sequence variants: a joint consensus recommendation of the American College of Medical Genetics and Genomics and the Association for Molecular Pathology. Genet Med. 2015 May;17(5):405–24. https://doi.org/10.1038/gim.2015.30. Epub 2015 Mar 5. PMID: 25741868; PMCID: PMC4544753.

^f^PolymiRTS Database 3.0: http://compbio.uthsc.edu/miRSNP/

^g^Tikka A, Metso J, Jauhiainen M. ANGPTL3 serum concentration and rare genetic variants in Finnish population. Scand J Clin Lab Invest. 2017;77:601–609.

## Discussion

We have studied the possible contribution of the gene encoding ANGPTL3 in the aetiology of FCHL. Our hypothesis was that some rare gain-of-function variants could have a major effect on the disease or, on the contrary, that common variants with minor effect on ANGPTL3 function could be in different frequency with respect to the general population. The results of our study do not support the first possibility, since the identified variants are not predictive of relevant functional changes in the protein. There are no previous *ANGPTL3* sequencing studies looking for GOF mutations in subjects with FCHL. At least 5 different *loci* have been associated with rare cases of monogenic FCHL: *LDLR*^16,17^, *LPL*^15^, *APOE*^34^, *PCSK9*^35^ and *APOA5*^36,37^, but *ANGPTL3* does not appear to be associated with this form of FCHL nor familial hypercholesterolemia (FH). Although FH and FCHL are different phenotypes, there is some degree of overlap between the two entities since they share many clinical aspects. Studies in subjects with genetic hypercholesterolemia of unknown origin suggestive of FH have also failed to detect causal mutations in *ANGPTL3*. We have not found any severe mutation neither in cases nor in controls in the total of 654 alleles investigated. This leads us to think about how well preserved is this gene probably related to the importance of this gene in human metabolism.

These results contrast with the role of ANGPTL3 in the lipid phenotype called familial combined hypolipidemia (FHBL2, OMIM #605019)^20^, in which LOF mutations in *ANGPTL3* are responsible of reduced plasma levels of TC, TG, VLDL cholesterol, LDLc, apoB, and free fatty acids, just the opposite lipid profile found in FCHL. Furthermore, FCHL and familial combined hypolipidemia share abnormal hepatic VLDL secretion rates as the main mechanism of the lipid abnormalities, being increased in FCHL^38,39^ and decreased in familial combined hypolipidemia^40^.

Most cases of FCHL are considered as a complex disease with interaction of polygenes or multiple allele relationships with effect on TC, TG and environmental factors, mainly obesity and diets rich in saturated fat. *ANGPTL3* genetic variation has not been associated with FCHL or mixed hyperlipidemia in genome-wide association studies (GWAS)^13,41^. Similar conclusions can be drawn from large-scale deep-coverage whole-genome sequencing^42^. Our study cannot rule out that the genetic variation in *ANGPTL3* participates in the final phenotype of polygenic forms of FCHL. We found four variants with different allele frequency in FCHL subjects and in normolipemic controls: c.607-109T>C, c.607-47_607-46delGT, c.835+41C>A and c.*52_*60del. The first three are located in intron regions and the in silico analysis does not predict any splicing change with clinical significance, so their contribution to FCHL seems unlikely. The variant c.*52_*60del, located in 3′UTR, presented statistically significant differences in allelic frequencies between FCHL subjects and normolipemic controls: 0.276 and 0.102, respectively (P < 0.001). This variant has been previously associated with two microRNAs, hsa-miR-151a-3p and hsa-miR-7702, modulators of gene expression^32^. However, this is a very frequent genetic variant in the general population and this variation has not been previously associated with cholesterol and triglyceride concentrations^43,44^, so its implication in the FCHL pathogenesis is unlikely, although it should be confirmed in future studies.

In summary, no GOF mutations in *ANGPTL3* were present in a large group of unrelated subjects with FCHL. Our results do not support a substantial role of *ANGPTL3* in FCHL.
